# Physical Fitness and Dietary Intake Improve Mental Health in Chinese Adolescence Aged 12–13

**DOI:** 10.3389/fnint.2022.921605

**Published:** 2022-07-11

**Authors:** Wenjie Liang, Jian Fu, Xin Tian, Jiaxue Tian, Yu Yang, Wencui Fan, Zijuan Du, Zheyu Jin

**Affiliations:** ^1^College of Physical Education, Yangzhou University, Yangzhou, China; ^2^Xi’an International Studies University, Xi’an, China

**Keywords:** cardiorespiratory fitness, calcium intake, mental health, adolescents, physical fitness

## Abstract

**Background:**

Mental health has become a major public health issue worldwide. Biological and epidemiological studies have suggested that diet and physical fitness play a role in the prevention or cure of mental disorders. However, further research is required to elucidate the relationship between diet, physical fitness, and mental health. The study aims to provide a theoretical basis for promoting an adolescent healthy lifestyle and preventing mental problems by exploring the relationship between physical fitness, calcium intake, calorie intake, and adolescent mental health.

**Methods:**

A cross-sectional study of a sample of adolescents (*N* = 253, 12–13 years) was conducted. The study involved adolescents from three middle schools in Central Jiangsu Province, including 136 boys and 117 girls. Weight, height, and body mass index were measured. Physical fitness was scored using the Chinese National Student Physical Fitness Standard. Diet data were collected using a weighed 7-day food diary to estimate energy intake and dietary calcium intake. The mental health status of the participants was assessed using the Chinese Middle School Student Mental Health Scale. A *T*-test and analysis of variance were used to analyze the differences of variables between different genders and body mass index, and Pearson correlation and stepwise multiple regression were used to explore the relationship between physical fitness, dietary intake, and mental health.

**Results:**

The height (165.13 ± 8.07), weight (55.24 ± 13.00), and strength quality (64.93 ± 21.66) of boys are higher than those of girls (161.67 ± 6.44,48.99 ± 8.97, 58.40 ± 23.75, *P* < 0.05), and the flexibility quality (74.59 ± 14.75) of girls is higher than that of boys (68.30 ± 20.84) (*P* < 0.05). There were significant differences in the total scores of speed and physical fitness (F values were 4.02187.73, 3.07, 5.95, 10.33, and 9.52, respectively, *P* < 0.05). There was a significant positive correlation between calcium intake, cardiopulmonary fitness, and mental health (*r* = 0.276, *P* < 0.01; *r* = 0.159, *P* < 0.05). Calcium intake and cardiopulmonary fitness could explain 8.4% of the changes in the mental health of adolescents aged 12–13(ΔR_2_ = 0.084, *P* < 0.05).

**Conclusion:**

Adequate calcium intake and the improvement of cardiopulmonary fitness in adolescents aged 12–13 are essential for the good development of their mental health. Future research in this field should examine the prospective associations between multiple measures of physical fitness composition and other nutrients ingested and mental health outcomes, as well as intervention studies that seek to provide evidence of causality.

## Background

Mental health problems affect 10–20% of children and adolescents worldwide (Kieling et al., 2011). Childhood and adolescence represent periods of rapid growth and brain development characterized by neuronal plasticity, formulation of self-concept, and the establishment of behavior patterns that may influence mental health (Lubans et al., 2016). Good physical fitness and a reasonable and balanced dietary structure are the implicit and explicit resources to form a healthy lifestyle and behavior, and they are also the main risk factors to determine current and future mental health. Considering the negative consequences of adolescent mental health problems, it is very important to find effective prevention methods to improve adolescent mental health.

Physical activity is an effective tool to improve health. Many scholars have discussed the mode, time, intensity, and mental health of physical activity from several points of view. There is evidence suggesting that physical activity is a protective factor against mental health problems such as depression (Mammen and Faulkner, 2013). Although there is no clear consensus, neurobiological, psychosocial, and behavioral mechanisms have all been hypothesized to explain the relationship between physical activity and mental health (Lubans et al., 2016). However, the level of physical activity of each person fluctuates at any time under the influence of society, family, and individual motivation. Physical fitness is the ability of the body to perform physical activity (Rodriguez-Ayllon et al., 2018). Although physical fitness is influenced by genetics (Fisher et al., 2015) to a certain extent, it represents the type, frequency, intensity, and duration of physical activity over time (Blair et al., 2001). Therefore, physical fitness is a symbol of the aftereffect of physical activity, and also provides a more stable measurement standard for the level of regular physical activity. Also, compared with physical activity, physical fitness is more predictive of health outcomes in adolescents (Bovet et al., 2007). Given the relationship between physical activity and physical fitness, the mechanisms proposed to explain the relationship between physical activity and mental health might also apply for physical fitness and mental health (Silverman and Deuster, 2014). The health-related components of physical fitness are commonly classified as cardiorespiratory fitness, muscular strength and endurance, flexibility, and speed (Ministry of Education of the People’s Republic of China, 2012). Many studies have been conducted from one or a combination of two aspects of physical fitness. Cardiorespiratory fitness refers to the capacity of the circulatory and respiratory systems to supply oxygen to the skeletal muscle mitochondria for energy production during physical activity (Pillsbury et al., 2013). Cardiorespiratory fitness can influence mental health outcomes through neurobiological processes (Silverman and Deuster, 2014). Studies have shown that cardiopulmonary health is positively correlated with the mental health of adolescents, (Greenleaf et al., 2010; Martin et al., 2011) and lower cardiorespiratory fitness shows higher levels of depression. Likewise, adequate muscular fitness has been shown to be associated with high self-esteem (Kalogiannis and Papaioannou, 2007; Lubans and Cliff, 2011) and low levels of anxiety. Muscular fitness is the ability of the body to exert maximal force against an external resistance (i.e., muscular strength) (Raghuveer et al., 2020). Muscular strength may also depend on cultural norms (Rodgers et al., 2012), thereby possibly affecting mental health through sociocultural mechanisms. Many researchers have explored the relationship between physical fitness and mental health, but they still have no clear consensus. The importance of speed to teenagers’ mental health has not been paid attention to in previous literature. We also do not know whether one component of physical fitness is more important than others, and the correlation intensity between various physical fitness and different dimensions of adolescent mental health is different, which still needs to be further discussed.

Diet, as an important variable to intervene in mental health, is another focus of this study. There is increasing evidence that certain dietary patterns have a positive impact on health. A healthy, balanced diet includes a variety of vegetables and fruits, whole grains, seafood and nuts, moderate amounts of low-fat dairy, red meat, and saturated and *trans* fats, all of which have been associated with good mental health (Hosker et al., 2019). The more western fast-food or highly processed food you eat, the more likely you are to develop psychological symptoms such as depression and anxiety (Dimov et al., 2019). There is consistent evidence of an association between unhealthy diets and worse mental health in children and adolescents, but more research is needed for better conclusions (O’Neil et al., 2014). Recently, more attention has been paid to the association between trace mineral intake and mental health. Studies have shown that low intake of zinc, copper, and manganese was associated with depression and anxiety symptoms (Nakamura et al., 2019). Similarly, calcium supplementation is more effective in reducing the risk of depressive symptoms (Wu et al., 2020). Calcium has the biological function of regulating the synthesis and release of neurotransmitters in the nervous system and plays an important role in activating neurons and regulating moods (Marambaud et al., 2009; Pal, 2021). Additionally, calcium is required to produce serotonin, which is the precursor of melatonin (Nongonierma and FitzGerald, 2015). Melatonin plays a fundamental role in sleep regulation and the maintenance of emotional health (Zisapel, 2018). These tiny improvements in dietary habits and nutrient intake lead to major benefits for adolescent mental health and development. Many research reports have explored the relationship between dietary patterns, frequency of intake of vegetables and fruits, intake of minerals and vitamins, and mental health at different levels, but the unavoidable question of studying the relationship between diet and mental health in children and adolescents is how to apply better dietary survey methods. Whether it is a 24-h review method, 3-day weighing record method, or food frequency questionnaire method, each has its own advantages and disadvantages, and there are still inconsistencies and gaps in the evidence obtained. Due to synergistic effects between nutrients (Hu, 2002), some scholars suggested a measure of overall diet as a better indicator in studies of the effects of diet quality on mental health. Taking advantage of the particularity of students’ unified eating, our study investigates and calculates the calorie and calcium intake of diet by combining the recording and weighing method. The purpose is to understand the dietary patterns and habits of the respondents without reducing the reliability of information, so as to better reveal the relationship with mental health.

Childhood and adolescence are likely to be critical times for establishing good mental health but there is still much to be learned about factors that may have a positive impact on psychological health in childhood (Sawyer et al., 2012; Lubans et al., 2016). Diet and physical fitness have been paid special attention as important factors affecting mental health, but the underlying mechanism of their interaction is still unclear. Based on the above analysis, our research will further clarify and discuss the relationship between the three and provide methods and theoretical basis for adopting reasonable dietary behaviors and physical exercise to control and prevent adolescents’ mental health problems. We hypothesized that: (1) different components of physical fitness are differently associated with mental health and (2) lower calcium intake and cardiorespiratory fitness both predict lower levels of mental health.

## Materials and Methods

### Design and Participants

The current study selected participants based on relevant research and adopted stratified random sampling from three middle schools in Central Jiangsu Province (China). Schools were selected for their representativeness, accessibility to our research team, and availability of the teachers to assist with logistics. Only students aged 12 and 13 were invited to participate in the study (*n* = 302). A written informed consent from their parents or guardians was received. All participants received comprehensive medical screening, and students with the following diseases are excluded: (1) cardiovascular or respiratory diseases; (2) bone, muscle, and ligament injuries or a history of such injuries; (3) surgical history of major diseases; (4) history of mental disorder or nervous system disease. A total of 277 adolescents were eligible for the study. Before data collection, all participants were informed about the purpose of the study and that they could withdraw at any time. Each school has 2 research assistants who provide guidance on the completion of questionnaires and conduct physical fitness testing. The participants were tested at their respective schools in the winter of 2020 during school time. All the testing personnel received the same training beforehand to make sure there were no discrepancies in how the tests were carried out. Among these participants, 24 did not complete the testing protocol. Eventually, only 253 adolescents (136 boys and 117 girls) were included in the data assessment. The study was conducted in accordance with the recommendations of the World Medical Association’s Declaration of Helsinki and approved by the Institutional Review Committee of Jinhu Hospital of Traditional Chinese Medicine (JHZYY202010).

### Measurements

#### Anthropometry

The body height and weight of the subjects were measured through a height and weight tester (HK6800-ST, China). During the test, the subjects were barefooted and wearing only thin clothing. Body mass index (BMI) was measured as a covariate because it is correlated with both dietary intake and mental health measures included in the analyses. Body mass index (BMI) was calculated as weight divided by height squared (kg/m^2^), and “School-age Children and Adolescents Screening Standards for Overweight and Obesity” (WS/T 586-2018) (National Health and Family Planning Commission of the People’s Republic of China, 2018) was adopted to divide the body mass index into four grades: low weight, normal weight, overweight, and obesity.

#### Physical Fitness Assessment

Physical fitness was measured using the Chinese National Student Physical Fitness Standard (Zhu et al., 2017). Primary outcome measures the following: the cardiorespiratory fitness test, which measured an individual’s aerobic capacity, vital capacity, and mid-distance running (800 m for girls and 1,000 m for boys), was used to measure cardiorespiratory fitness. Muscle fitness assessment adopted lower extremity muscle strength (standing long jump) to test the body’s ability to exert maximum force against external resistance. Speed was assessed by the 50-m run test, which reflected the ability to move quickly on the ground or move the limbs quickly. Flexibility refers to an individual’s range of motion around a joint or group of joints, and the flexibility test used in this research was the sitting forward flexion test.

Vital capacity was assessed by the FHL-II spirometer (Xindong Huateng Inc., Beijing, China). After the first maximum exhalation, participants rested for a minute before the second test, and the best performance was recorded.

Men’s 1,000-m/women’s 800-m tests were conducted on the 400-m track. Before the test, students are required to do a full warm-up and then they started in a group of 10–12 people by using a standing start.

The standing long jump was tested on a bunker with the sand surface level with the ground or on a flat ground with soft soil using a measuring ruler. The subject’s feet were naturally separated, and after standing on the jumping line, both feet took off at the same time. Records were in centimeters to one decimal place. The test was taken three times and the best score was considered.

The 50-m running test was conducted on the track and field. The subjects were in groups of 2-3 and started standing up; when they heard the start signal, they immediately started and ran to the finish line with all their strength. Test equipment: starting flag, starting whistle, and stopwatch.

The sitting body flexion test was performed with an electronic sitting flexion tester (Wanqing WTS-600, Shanghai, China). The test subjects were asked to sit on the test plate, with their legs straight, their feet flat on the test longitudinal plate, their upper bodies flexing forward, their arms stretching forward, and the Vernier was gradually pushed forward with their middle fingertips until they could not be pushed forward. Records were in centimeters to one decimal place.

Finally, according to the “National Student Physical Health Standards” promulgated by the Ministry of Education and the General Administration of Sports of the People’s Republic of China, the students’ scores were converted into corresponding scores.

#### Mental Health Assessment

Mental health was tested by the mental health scale for middle school students (Su and Huang, 2007) prepared by Sudan and Huang Xiting. It has high reliability (retest reliability is 0.82) and is widely used in China. The scale was a multi-dimensional measuring tool. It evaluated the mental health of middle school students from five aspects: life, study, interpersonal communication, examination, and emotion. There were 25 items in total, and the 5-level score was adopted. The higher the score, the better the mental health. We took the school as a unit for group measurement, and the testers in the classroom uniformly distributed the mental health scale. At the beginning of the test, the test subjects were instructed to fill in the questionnaire with unified and clear instructions, and the questionnaire was collected immediately after completion.

#### Dietary Intake Assessment

The study used a dietary survey method combining the food recording method and the weighing method. The daily dietary situation was reflected through a cross-sectional dietary survey, and the dietary intake of the participants was recorded for 7 consecutive days, including 5 days on-campus and 2 days off-campus. The research subjects filled in the name, category, quantity, and weight of the food they ingested according to the “Daily Diet Log” (Egger and Flueck, 2020; Tian et al., 2021). A 2-day pre-investigation was conducted before the formal investigation. The investigator explained the filling requirements to the students according to the school’s fixed recipes and quantitative cutlery and promptly corrected and answered the questions that the subjects had during the recording process. During the formal investigation, each class was assigned an investigator for timely observation and guidance. After the end of the day’s investigation, the investigator collected the information, checked and corrected the information, and distributed the information the next day. It could well make up for the inaccurate estimation of food intake caused by the 24-h review method for recalling information. Finally, the different meals provided by the respondents were weighed and recorded. On rest days, the respondents and their guardians would record the dietary intake for the 2 days, and the investigator would provide online Q&A to ensure the reliability of the survey. A total of 253 valid questionnaires were returned. The calcium intake and calorie intake in the diet were calculated according to the “Chinese Food Composition Table (2019 Edition).”

### Statistical Analysis

SPSS 20.0 (SPSS Inc., Chicago, IL, United States) was used to analyze the data. The general demographic data, factors of physical fitness, dietary intake, and mental health levels of the research subjects were analyzed by descriptive statistics, the quantitative data were described by the mean ± standard deviation, and the data were tested for normality. The binary variable sex and each variable were used for *t*-test, the multi-category variable was used to replace the continuous variable, and ANOVA was used to analyze whether there was a difference between the different body mass indexes of adolescents and each variable. The study also used Pearson correlation and stepwise multiple regression for statistical analysis. The level of statistical significance was set at *P* < 0.05 (two-tailed). Graphpad Prism8.0.2 software was used for graphics processing.

## Results

### Comparison of Mental Health, Diet, and Physical Quality in Different Genders and BMI

Among the 253 adolescents aged 12–13 who participated in the survey from November 2020, the average mental health score was (72.7 ± 9.05), the average calcium intake was 2,241.87 ± 694.17 mg, and the average caloric intake was 36,528.18 ± 7,956.8 KJ. The average scores of cardiorespiratory fitness, muscle fitness, flexibility, speed, and total physical fitness were (131.44 ± 33.11), (61.91 ± 22.84), (71.01 ± 20.38), (75.95 ± 17.53), and (340.30 ± 62.10), respectively. Boys’ height, weight, BMI, and muscle fitness were higher than girls’ (*P* < 0.05), and girls’ flexibility was higher than boys’ (*P* < 0.05), and other variables had no significant differences between genders. There was no significant difference in mental health scores among different BMIs, while the differences in total scores of height, weight, BMI, calorie intake, muscle fitness, speed, and physical fitness were statistically significant (*p* < 0.05). The calcium intake, calorie intake, cardiorespiratory fitness, and muscle fitness of low-weight adolescents were higher than those of normal adolescents. Table 1 showed the comparison of psychological quality, diet, and physical fitness under different gender and BMI.

**TABLE 1:** Comparison of mental health, diet and physical fitness under different gender and BMI (mean ± SD).

		Gender (x ± s)			BMI (x ± s)					
Variable	Total (*n* = 253)	Male (*n* = 136)	Female (*n* = 117)	*P*	Low Weight (*n* = 23)	Normal (*n* = 168)	Overweight (*n* = 38)	Obesity (*n* = 24)	P	F
Mental health	72.70 ± 9.05	71.71 ± 9.66	73.85 ± 8.17	0.056	74.61 ± 9.61	73.03 ± 9.04	71.05 ± 8.64	71.17 ± 9.12	0.364	1.07
Height (cm)	163.53 ± 7.55	165.13 ± 8.07	161.67 ± 6.44	0.001	161.87 ± 8.26	162.76 ± 7.41	165.61 ± 6.39	167.27 ± 8.11	0.008	4.02
Weight (kg)	52.35 ± 11.71	55.24 ± 13.00	48.99 ± 8.97	0.001	38.96 ± 4.14	48.45 ± 6.34	62.99 ± 4.61	75.63 ± 10.89	0.001	187.73
BMI (kg/m^2^)	19.46 ± 3.50	20.11 ± 3.79	18.69 ± 1.97	0.001	14.83 ± 0.53	18.24 ± 1.57	22.95 ± 0.81	26.91 ± 2.16	0.001	383.42
Calcium intake (mg/week)	2,241.87 ± 694.17	2,313.59 ± 720.24	2,158.50 ± 655.81	0.074	2,371.20 ± 655.36	2,205.20 ± 670.10	2,278.19 ± 757.12	2,317.09 ± 807.28	0.650	0.55
Caloric intake (KJ/week)	36,528.18 ± 7,956.80	37,090.43 ± 8,328.52	35,874.62 ± 7,599.01	0.226	37,765.24 ± 8,164.55	35,743.79 ± 7,662.03	36,574.13 ± 7,933.80	40,760.68 ± 8,812.48	0.029	3.07
Cardiorespiratory fitness	131.44 ± 33.11	129.29 ± 32.87	133.94 ± 33.34	0.266	134.71 ± 24.51	132.88 ± 33.60	134.14 ± 31.94	113.87 ± 35.18	0.055	2.57
Muscle fitness	61.91 ± 22.84	64.93 ± 21.66	58.40 ± 23.75	0.024	67.09 ± 17.44	64.73 ± 21.60	55.75 ± 24.23	46.92 ± 26.66	0.001	5.95
Flexibility	71.01 ± 20.38	68.30 ± 20.84	74.59 ± 14.75	0.022	71.43 ± 16.22	72.75 ± 19.96	68.83 ± 18.11	61.83 ± 27.56	0.087	2.21
Speed	75.95 ± 17.53	77.12 ± 19.58	74.59 ± 14.75	0.244	75.22 ± 18.47	78.76 ± 15.50	74.87 ± 10.68	58.65 ± 27.18	0.001	10.33
Physical fitness	340.30 ± 62.10	339.63 ± 66.71	341.09 ± 56.55	0.851	348.45 ± 48.15	349.14 ± 57.78	333.59 ± 55.03	281.27 ± 81.11	0.001	9.52

### Correlation Analysis of Physical Fitness, Dietary Intake, and Mental Health

**FIGURE 1:**
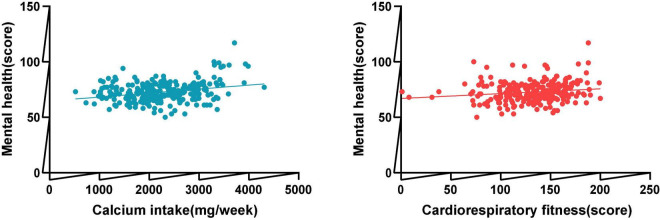
The correlation between dietary calcium intake, cardiopulmonary quality and mental health.

First of all, as shown in Table 2, the psychological health of the research subjects was significantly positively correlated with calcium intake and cardiorespiratory fitness (Figure 1), and the correlation with calcium intake was the greatest (*p* < 0.01). Secondly, there was a low correlation between physical fitness and mental health (*r* = 0.114), but it was not significant. Among the independent variables, there was a significant low correlation between calcium intake and cardiorespiratory fitness (*r* = 0.126, *P* < 0.05), and cardiorespiratory fitness was moderately positively correlated with muscle fitness and speed (*P* < 0.01).

**TABLE 2:** Correlation between physical fitness, dietary intake, and mental health (r).

Variable	Mental health	Caloric intake	Calcium intake	Cardiorespiratory fitness	Muscle fitness	Flexibility	Speed	Physical fitness
Mental health	1							
Caloric intake	0.004	1						
Calcium intake	0.276**	0.436**	1					
Cardiorespiratory fitness	0.159*	–0.040	0.126*	1				
Muscle fitness	0.054	–0.026	0.096	0.340**	1			
Flexibility	–0.04	0.060	–0.080	0.049	0.018	1		
Speed	0.081	–0.011	0.043	0.346**	0.545**	0.113	1	
Physical fitness	0.114	–0.014	0.089	0.772**	0.709**	0.393**	0.705**	1

**P < 0.001, *P < 0.05, the results are rounded to 3 decimal places.

### Regression Analysis of Mental Health, Calcium Intake, and Cardiopulmonary Fitness

**FIGURE 2:**
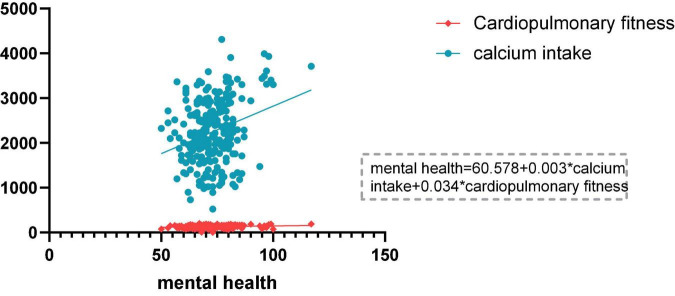
Regression curves and equations of dietary calcium intake, cardiorespiratory fitness, and mental health.

In order to further understand the relationship between adolescents’ physical fitness, dietary intake, and mental health with mental health as the dependent variable, a stepwise multiple regression analysis was performed on calcium intake and cardiorespiratory fitness (Table 3 and Figure 2). The two independent variables of calcium intake and cardiopulmonary fitness entered the final model. Calcium intake and cardiorespiratory fitness had a significant positive predictive effect on mental health, and they could explain 8.4% of the variation in mental health (*P* < 0.01).

**TABLE 3:** Regression analysis of mental health, calcium intake, and cardiopulmonary fitness.

Dependent variable	Constants and variables	B	Beta	t	F	R^2^	Δ R^2^	P
Mental health	(Constant) Calcium intake (Constant)	64.639 0.004 60.578	0.276	34.842 4.548 22.528	20.682	0.076	0.072	0.000**
	Cardiopulmonary fitness	0.034	0.126	2.074	12.627	0.092	0.084	0.039*
	Calcium intake	0.003	0.260	4.279				0.000**

**P < 0.001, *P < 0.05, the results are rounded to 3 decimal places.

## Discussion

Physical fitness is widely recognized as a power marker of health-related outcomes and an important determinant of current and future mental health. Numerous studies have shown a positive correlation between physical fitness and mental health (Loprinzi et al., 2017; Oliveira et al., 2019). Based on the cross-sectional study, we observed that there was a low correlation between total physical fitness and mental health in youth, and there was no significant level. However, the survey lacks information related to sensitive quality, which is not enough to draw powerful and clear conclusions. In the follow-up study, it is necessary to increase the sample size and investigate all physical fitness indicators so as to better evaluate the relationship between the two. But there is a significant positive correlation between cardiorespiratory fitness and the overall mental health of adolescents. The study also complements previous findings, suggesting that good cardiorespiratory fitness in adolescents leads to better mental health (Reddon et al., 2017). Although, longitudinal studies are lacking, cardiorespiratory fitness appears to be an important predictor of current and future mental health status. The relationship between cardiorespiratory fitness and mental health has been investigated in most studies. In recent years, more and more attention has been paid to investigate the relationship between muscle strength and mental health in adolescents (Smith et al., 2014; Appelqvist-Schmidlechner et al., 2020). Our study did not find a significant correlation between muscular strength and mental health. One possible explanation is that there are few and incomplete indicators for measuring muscular strength. Comprehensive and detailed measurements of core strength, upper body strength, and grip strength of adolescents are required. In adolescents, however, muscular strength and speed are mainly associated with appearance-related mental health outcomes, such as self-perception, perceived physical appearance, or physical self-worth (Cadenas-Sanchez et al., 2021). Differences in psychometric tools also make it difficult to compare research results horizontally. Of note, although no significant correlation was found between speed quality and mental health, the correlation coefficient of speed quality was more closely related to mental health than strength and flexibility. Therefore, future research on the relationship between physical fitness and mental health should include different measures of mental health factors to better understand whether components of physical fitness are associated with specific outcomes of mental health. In addition, there are few studies on the relationship between flexibility and mental health. Some studies believe that the improvement of flexibility can help patients with developmental delays to improve their psychological state (Shell et al., 2018). Our results show that flexibility cannot predict the mental health of adolescents aged 12–13, and the relationship between flexibility and mental health of different ages and specific populations is not well defined.

With changes in lifestyle, the number of overweight and obese children and adolescents increases substantially. The impact of obesity and diet quality on mental health has been of concern and supported by a diverse evidence base (Adan et al., 2019). It is logical that food intake and food quality can have an impact on brain function, which makes diet an adjustable variable for mental health, mood, and cognitive function (Dinan et al., 2018). The relationship between calcium intake and mental health in the literature is rarely studied and controversial. The significant positive association between calcium intake and mental health in adolescents observed in the present study is broadly similar to the results of previous studies (Miki et al., 2015; Wu et al., 2020). A previous Korean study (Kim et al., 2015) that investigated the relationship between calcium intake and mental health showed controversial results that increasing the frequency of calcium intake did not reduce the risk of mental illness. The inconsistent results of the study may be attributed to the methods in the study and the differences in gender, age, and race, which are related to the control variables. But our results provided positive evidence for an association between supplemental calcium intake and mental health in adolescents. In addition, we also observed that there was no correlation between BMI and mental health, and the change in adolescent BMI after classified adjustment of BMI did not have a significant impact on mental health. This is because overweight and obesity may be an important confounder in the relationship between diet and mental health (Halfon et al., 2013). Luppino et al. (2010) found an association between overweight and depression in adults, but not in those under 20. The results of this study showed no association between BMI and mental health in 12- to 13-year-olds, so this suggests that different relationships may emerge between different age groups. The imbalance between energy intake and energy expenditure is the direct cause of individual overweight or obesity. There are significant differences in caloric intake among different body mass indices in the study results, but there is no correlation between caloric intake and mental health, which better increases the reliability of the results of this study. Essential nutrients that have the potential to have beneficial effects on mental health must be obtained from the diet. Our study used the overall diet as a better indicator to investigate calcium intake and calorie intake from food sources. In the future, it is necessary to increase the frequency of different food intake and information on other nutrients and trace elements, and investigate people of different ages more accurately explain the relationship between diet and mental health.

Overall, our findings suggested that cardiorespiratory fitness and calcium intake were positively associated with better mental health. Further, stepwise multiple regression analysis showed that cardiorespiratory fitness and calcium intake could jointly explain 8.4% of the variation in mental health among adolescents aged 12–13. This study highlighted the importance of dietary calcium intake and cardiorespiratory fitness in adolescence and its potential role in modifying mental health over the life course.

Several limitations in this study should be discussed. First, there are significant gender differences in the development of adolescent physical fitness. We found that the strength and BMI of 12- to 13-year-old boys were greater than that of girls, and the flexibility of girls was better than that of boys. However, we did not highlight the relationship between gender and individual variables. In studies of specific components of physical fitness and mental health, the respective relational effects of adolescent boys and girls need to be considered. Secondly, in the dietary survey, we only analyzed the dietary calcium intake of one nutrient, thus lacking a comprehensive evaluation of the synergistic effect of other nutrients and calcium. Given the prevention of mental health problems and the critical period of growth and development, it is essential for adolescents to supplement dietary calcium intake and improve cardiopulmonary fitness. However, care is needed with calcium supplementation as it may be a risk factor for vascular disease (Tankeu et al., 2017). Consistent adverse effects on cardiovascular health have not been demonstrated for dietary calcium (Reid et al., 2017), and dietary calcium intake was lower in the survey sample. Therefore, we encourage getting calcium from the diet. In addition, it is worth noting that as a cross-sectional study, we do not know whether insufficient calcium intake leads to poor mental health, whether adolescents with poor mental health consume less calcium-rich foods, or whether the effect of cardiorespiratory fitness is an innate condition-determined, all of which imply that genetics and family environment may play a role. Therefore, there is an urgent need for longitudinal studies and randomized controlled trials investigating the explanatory mechanisms between physical fitness, diet, and mental health outcomes, especially in adolescent populations. As most mental health problems first manifest in adolescence and early adulthood, it is necessary to fully understand the relationship between relevant variables and determine the short-term and long-term effects of physical fitness and diet on adolescents’ mental health so as to provide a more accurate theoretical basis for adolescents’ good mental health and healthy lifestyle in China.

## Conclusion

The cross-sectional analysis showed gender differences in muscular strength and flexibility, and differences in caloric intake, muscular strength, speed, and overall physical fitness between BMIs among adolescents aged 12 and 13. Through the analysis of the correlation between dietary intake, physical fitness, and mental health of adolescents, the following conclusion was reached in this study. There was a positive correlation between cardiorespiratory fitness and calcium intake, and higher cardiorespiratory fitness and adequate calcium intake were significantly associated with lower levels of psychological distress. Strength, speed, flexibility, and mental health were not significantly associated. These findings suggested that adequate dietary calcium intake and improved cardiorespiratory fitness may lead to better mental health among teenagers aged 12 and 13 in China.

## Data Availability Statement

The original contributions presented in this study are included in the article/supplementary material, further inquiries can be directed to the corresponding author.

## Ethics Statement

The studies involving human participants were reviewed and approved by Jinhu Hospital of Traditional Chinese Medicine. Written informed consent to participate in this study was provided by the participants or their legal guardian/next of kin.

## Author Contributions

WL and JF: conceptualization and methodology. JT: software. JT, YY, and WL: validation. WL: formal analysis, resources, and writing—original draft preparation. WL, XT, JT, and YY: investigation. WF: data curation. ZJ: graphical processing of data. ZD: writing—review and editing. WL and XT: visualization. JF and WL: supervision and project administration. All authors have read and agreed to the published version of the manuscript.

## Conflict of Interest

The authors declare that the research was conducted in the absence of any commercial or financial relationships that could be construed as a potential conflict of interest.

## Publisher’s Note

All claims expressed in this article are solely those of the authors and do not necessarily represent those of their affiliated organizations, or those of the publisher, the editors and the reviewers. Any product that may be evaluated in this article, or claim that may be made by its manufacturer, is not guaranteed or endorsed by the publisher.
